# Eye-background contrast as a quantitative marker for pupal age in a forensically important carrion beetle *Necrodes littoralis* L. (Silphidae)

**DOI:** 10.1038/s41598-020-71369-0

**Published:** 2020-09-02

**Authors:** M. Novák, K. Frątczak-Łagiewska, A. Mądra-Bielewicz, S. Matuszewski

**Affiliations:** 1 Department of Ecology, Faculty of Environmental Sciences, Czech University of Life Sciences Prague, Kamýcká 129, 165 00 Prague, Czech Republic; 2 Laboratory of Criminalistics, Adam Mickiewicz University, Św. Marcin 90, 61-809 Poznań, Poland; 3 Wielkopolska Centre for Advanced Technologies, Adam Mickiewicz University, Uniwersytetu Poznańskiego 10, 61-614 Poznań, Poland; 4 Department of Animal Taxonomy and Ecology, Adam Mickiewicz University, Uniwersytetu Poznańskiego 6, 61-614 Poznań, Poland

**Keywords:** Entomology, Biological techniques, Developmental biology

## Abstract

Insect pupae sampled at a death scene may be used to estimate the post-mortem interval. The pupal age is however difficult to estimate, as there are no good quantitative markers for the age of a pupa. We present a novel method for pupal age estimation based on the quantification of contrast in intensity between the eyes of a pupa and the middle grey photography card as a standard background. The intensity is measured on a standardized scale from 0 (perfect black) to 255 (perfect white) using computer graphical software and pictures of the eye and the background taken with a stereomicroscope. Eye-background contrast is calculated by subtracting the average intensity of the eye from the average intensity of the background. The method was developed and validated using pupae of *Necrodes littoralis* (Linnaeus, 1758) (Coleoptera: Silphidae), one of the most abundant beetle species on human cadavers in Central Europe. To develop the model, pupae were reared in 17, 20 and 23 °C, with a total of 120 specimens. The method was validated by three raters, using in total 182 pupae reared in 15, 17, 20, 23 and 25 °C. We found a gradual increase in eye-background contrast with pupal age. Changes followed generalized logistic function, with almost perfect fit of the model. Using our method pupal age was estimated with the average error of 8.1 accumulated degree-days (ADD). The largest error was 27.8 ADD and 95% of age estimates had errors smaller than 20 ADD. While using the method, different raters attained similar accuracy. In conclusion, we have demonstrated that eye-background contrast is a good quantitative marker for the age of *N. littoralis* pupae. Contrast measurements gave accurate estimates for pupal age. Our method is thus proven to be a candidate for a reliable approach to age insect pupae in forensic entomology.

## Introduction

Insects visiting human cadavers are used for postmortem interval (PMI) estimation^1,2^. Most frequently, forensic entomologists use insect age as a basis for the minimum PMI estimation^3,4^. However, estimating the age of some insect life stages may be problematic. The pupa, for example, lacks reliable and easy to use quantitative age markers. This is particularly important, as a considerable portion of premature development takes place at this stage, at least 50% in the case of flies^1,5–7^ and at least 30% in the case of beetles^8–11^.

Pupae are frequently killed and preserved in alcohol during insect collection at a death scene. Various methods have been proposed to estimate the age for such specimens, mostly for puparia of forensically relevant blow flies. It has been suggested to analyze changes in cuticle composition^12–14^, gene expression^15–23^ or morphology and anatomy^24–33^. All these approaches have some advantages, which, however, are usually overshadowed by drawbacks, i.e. qualitative markers for age, changes of markers covering only a small portion of the pupal stage, high costs or time-consumption, destruction of specimens, expert knowledge or highly sophisticated and rare equipment necessary for the analysis.

Most methods developed to age forensically relevant pupae are based on morphological analyses^7,22,25,27–29,32,34–45^. Such analyses require only a stereomicroscope and are generally non-destructive. Their major disadvantage concerns the qualitative nature of these analyses. Morphological features enable the division of intra-puparial or pupal stage into only a few substages, delineated by developmental landmarks. Due to the complexity of the development, the substage-delineating landmarks are usually defined by a combination of multiple morphological features. The number of landmarks (and substages) recognized in case of the intra-puparial period of fly species can vary from six^37,42,44^ up to 13, with combinations of as many as 29 morphological features^28,32^. As a consequence, the specimen analysis requires an entomologist with expert knowledge in the morphology of immature insects and thus may be impractical in routine forensic casework.

Pigmentation of body extremities, bristles, claws and eyes change during pupal development. Shortly after pupariation changes in color have also been observed in a fly puparium^35,46^. The most profound changes appear at the end of the pupal stage and thus limit their use for age estimation. On the other hand, changes in eye coloration are gradual and have been observed to start early in the pupal stage of beetles or flies^36–38,44^.

Changes in eye color of phanerocephalic pupae and pharate adults have been analyzed in several studies describing morphological markers for the pupal age. Most of these works describe eye color using nominal terms (i.e. yellow, reddish etc.), which allow only for qualitative and subjective interpretation of these changes^7,22,27,29,34,36–45,47,48^. Only two studies attempted to categorize changes in the color of pupal eyes using standardized color charts, which enabled them to use a larger number of categories. Greenberg^35^ used color codes provided in a color atlas^49^ to distinguish 9 eye color categories in *Phormia regina* (Diptera: Calliphoridae) intra-puparial development under 22 °C and another 9 categories, mostly different, under 29 °C. However, the atlas is a printed book and therefore the perception of the color charts will depend on the light conditions in which they are observed. Moreover, because print fades over time, colors in an old book will differ from colors in a new one. Furthermore, entomologists may differ in their perception of the pupal eye color and these differences may result in different color codes assigned by different entomologists to the same insect specimens. Such problems may be partially resolved with the use of a digital color library, as in Brown et al.^28^. The authors distinguished 27 eye color categories in *Calliphora vicina* (Diptera: Calliphoridae) intra-puparial development under 22 °C, providing images of eyes photographed using the same blue background. These 27 color categories were grouped into five broader categories for pupal age estimation^28^. Therefore, the semi-quantitative color measure was finally transformed into a purely qualitative measure with only five categories. Another problem is that individual images from this study reveal differences in exposure (see Fig. 1 in Brown et al.^28^), which makes them difficult to compare, not only with each other, but also with future images of *C. vicina* pupae. Light-pigmented eyes of a young pupa in an underexposed picture may look the same as dark-pigmented eyes of an older pupa in an overexposed picture. For that reason, to reliably apply the method of Brown et al.^28^ in respect to the eye color categorization, it would be necessary to duplicate light conditions in which the reference pictures were taken.

**Figure 1:**
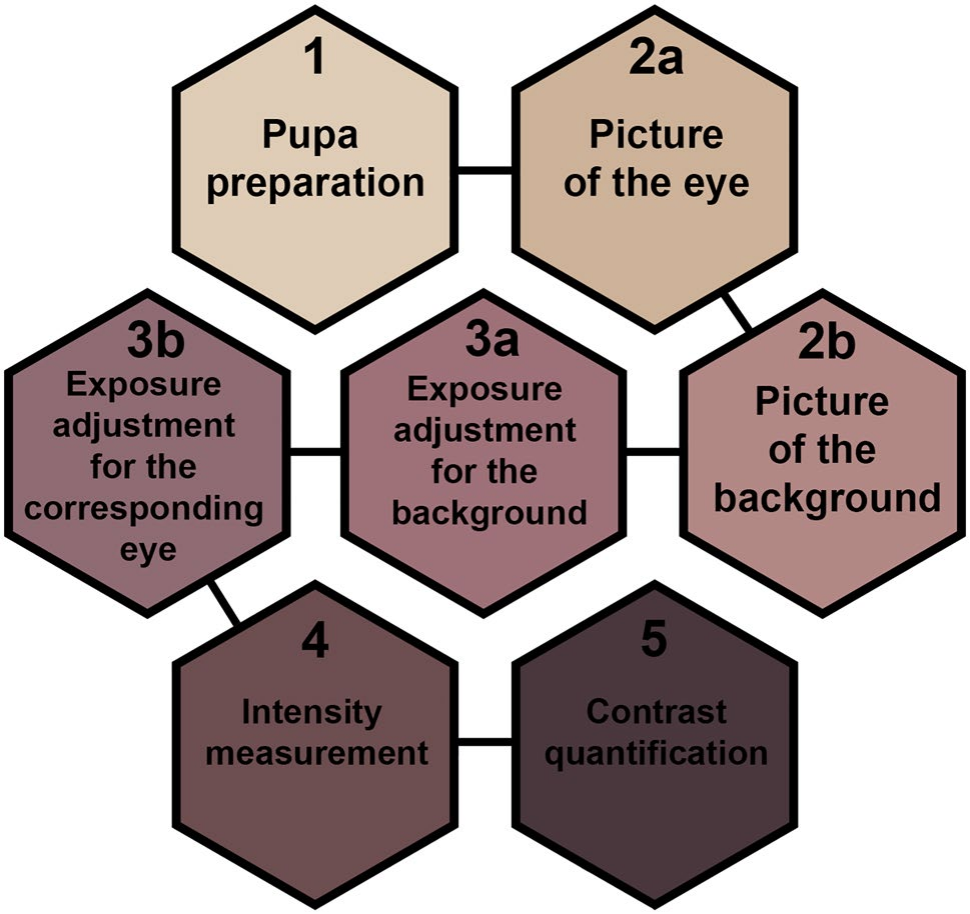
Flow chart representing stages of the method.

Despite the above disadvantages, changes in pupal eye pigmentation represent clear advantages. The pigmentation changes gradually throughout most of the pupal stage, thus providing high resolution and coverage of the stage. Moreover, these changes seem to be independent of environmental conditions. In addition, pigmentation of pupal eyes may be measured by everyone and no expert knowledge is necessary for this purpose. On the other hand, several improvements are necessary to make the most of this method’s potential. First, how to define and measure an insect’s eye color to get quantitative and reliable information? Second, how to remove the effect of changing light conditions?

In this work, we present a simple method that addresses the above-mentioned difficulties and finally enables accurate estimation of the pupal age based on measurements of eye pigmentation. We propose to quantify the contrast in intensity between the eye of a pupa and the grey standard background. We have developed a simple method for average RGB intensity quantification in pictures of the eye and the background using popular graphic software and standard stereomicroscopes. The method has been tested on pupae of *Necrodes littoralis* (Linnaeus, 1758), a species of carrion beetles with high importance in forensic entomology^8,50–55^.

## Outline of the method

Our method was inspired by the work of Matuszewski and Szafałowicz^56^, in which the authors proposed a simple way to quantify contrast in fingermarks and fingerprints. The authors suggested to define a contrast as a difference between the average intensity of pixels in ridges and valleys and proposed to quantify intensity using graphic software from scanned images of fingermarks or fingerprints.

Similarly, to get quantitative information about the pigmentation of pupal eyes we propose to quantify contrast between the intensity of the eye and the standardized background. The intensity of pixels in the eye and the background is measured in pictures taken with a stereomicroscope, using a standardized scale from 0 (perfect black) to 255 (perfect white). We propose to quantify the average intensity of red, green and blue pixels (i.e. RGB channel) using the in-built functions of popular graphic software (here Adobe Photoshop). To control light conditions, we propose to take pictures of the eye and the readily available middle grey (18% of grey) photography card as a background, under the same lighting conditions (the same lamp setup) while using the same stereomicroscope and camera settings. To achieve the best accuracy it is essential that the setup, including position of light sources, light diffuser etc., remains the same between images of the eye and the background that form a pair for which the contrast will be determined. The mid-tone grey card was chosen for the background, because its intensity values are approximately in the middle of the RGB intensity scale, leaving enough portions of the scale on its both sides for the exposure adjustment. Pairs of pictures are subsequently used to quantify eye-background contrast by subtracting the average RGB intensity of the eye from the average RGB intensity of the background. To control differences in contrast between picture pairs taken under different microscope exposure settings (lower exposure for young, very bright pupae and higher exposure for older, darker pupae), we propose to adjust exposure within the pair to meet the same predefined value for the background picture (in this particular study 150 intensity value).

**Figure 2:**
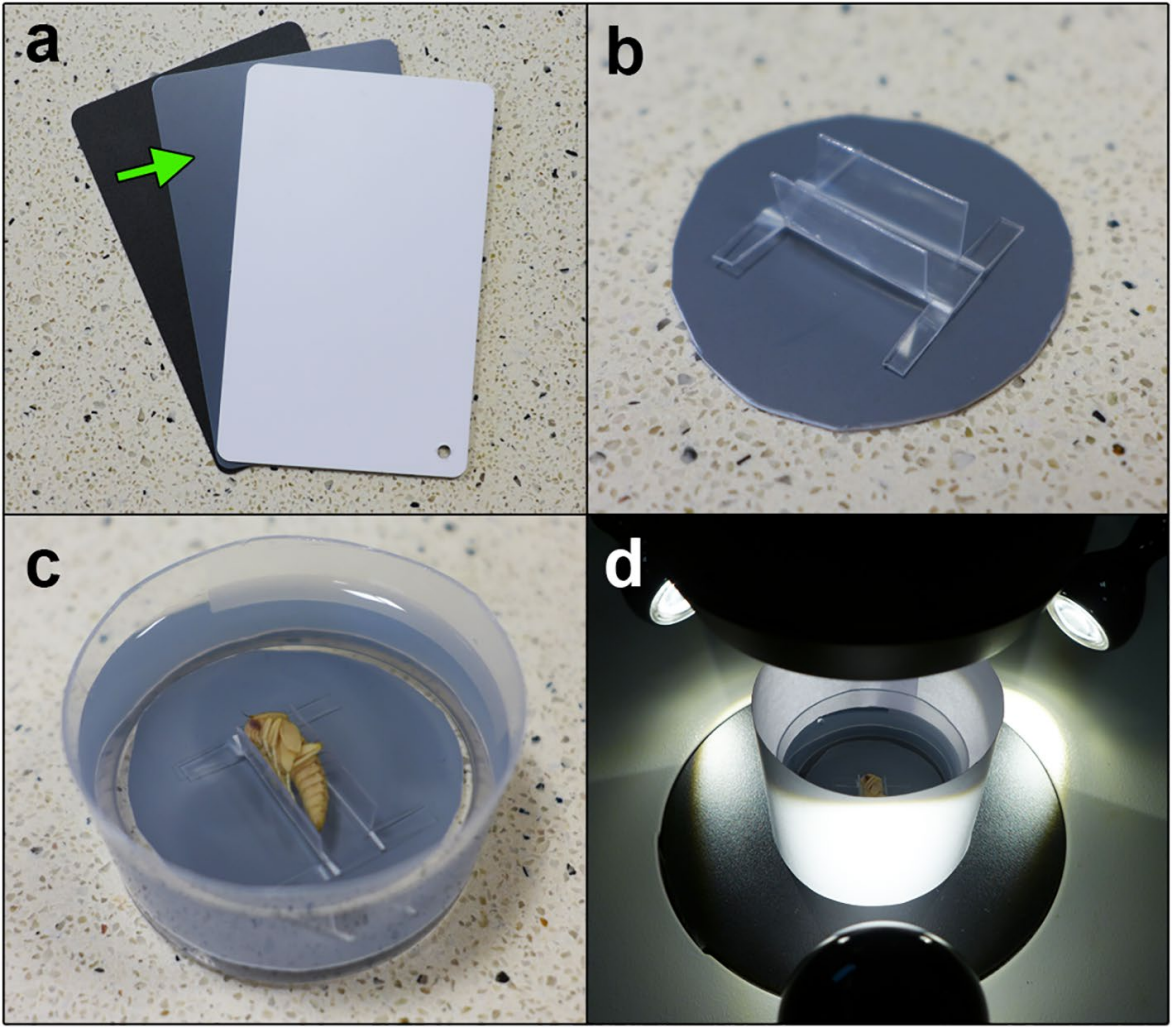
Technical details of the preparation and photographing of pupae. (**a**) middle grey photography card, (**b**) pupa holder on the grey photography card cut to fit the immersion-container, (**c**) holder with a pupa submerged in alcohol, (**d**) setup of immersion-container, light diffuser and lamps under a stereomicroscope objective.

**Figure 3:**
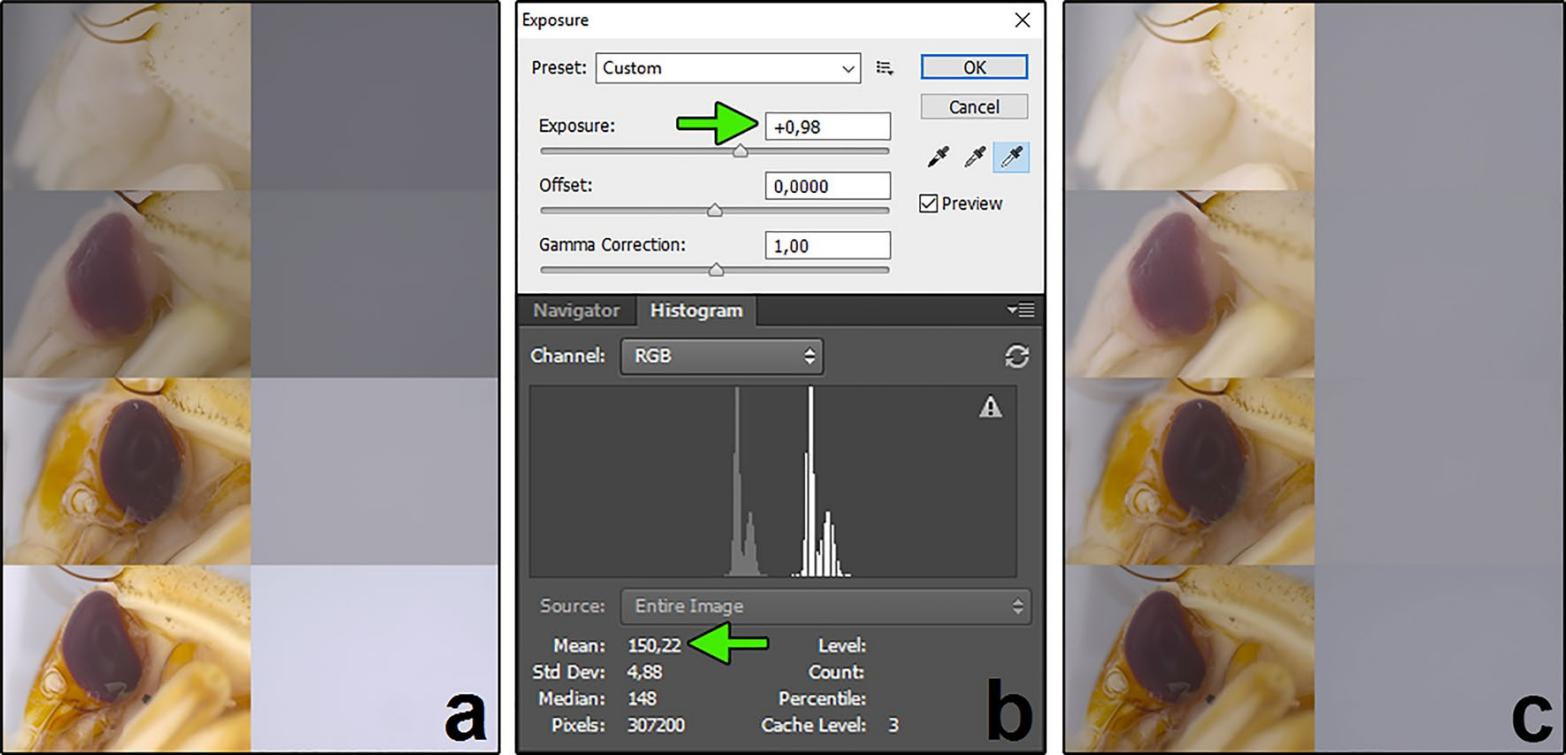
Details of the exposure adjustment. (**a**) picture pairs before the adjustment, (**b**) exposure and histogram screens, (**c**) picture pairs after the adjustment. Green arrow on the histogram screen points to the pre-specified value of the average RGB intensity (150). Green arrow on the exposure screen points to the exposure-change factor which should be applied to adjust exposure in the corresponding eye picture.

**Figure 4:**
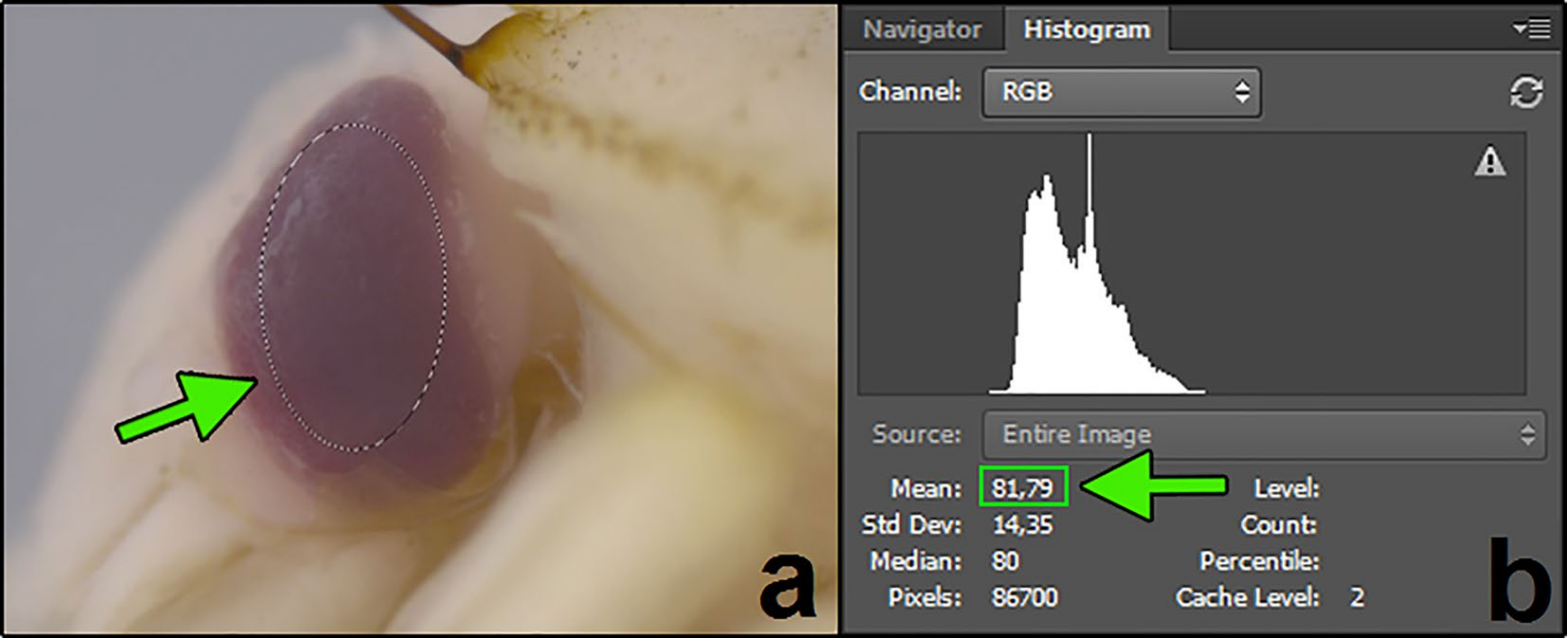
RGB intensity measurement in the eye picture (**a**). Green arrow on the histogram screen (**b**) points to the mean intensity value measured in the elliptical area pointed by green arrow on the eye picture.

The method consists of five steps: specimen preparation, photographing, exposure adjustment, intensity measurement and calculation of contrast (Fig. 1). We suggest preserving specimens according to current standards (e.g. near-boiling water for killing and fixation, 75% ethanol for storage)^57,58^. Because some pupae have transparent epidermis that covers the eyes, we recommend removing the epidermis and the adjacent antenna. This step is recommended for *N. littoralis*, but may not be necessary or even possible in other species. Specimens should be photographed in lateral view while immersed in alcohol using a light diffuser (Fig. 2). Care should be taken to avoid any shadows from the protruding pupal parts or the pupal holder. To take both pictures under the same light conditions, the pupa should be positioned on the grey background card and both pictures should be taken one after the other, with only minimal movement of the container between taking the pictures (Fig. 2). Afterwards, exposure adjustment is necessary. For this purpose, exposure in the picture of the background should be corrected to meet the pre-specified value of average RGB intensity, i.e. 150 in this study (Fig. 3). The correction will result in the exposure-change factor which should be subsequently applied to adjust exposure in the corresponding eye picture (Fig. 3). To quantify intensity, built-in tools of popular graphic software (here Adobe Photoshop) are used (Fig. 4). We suggest measuring intensity in the largest possible area of the eye using predefined software measurement tools. Since the average RGB intensity is calculated from a very large number of pixels (usually between 80 000 and 100 000), the technique is resistant to differences in the delineation of a measurement area. In the last stage, eye-background contrast is calculated.

## Results

### Model for the relationship between eye-background contrast and pupal age

**Figure 5:**
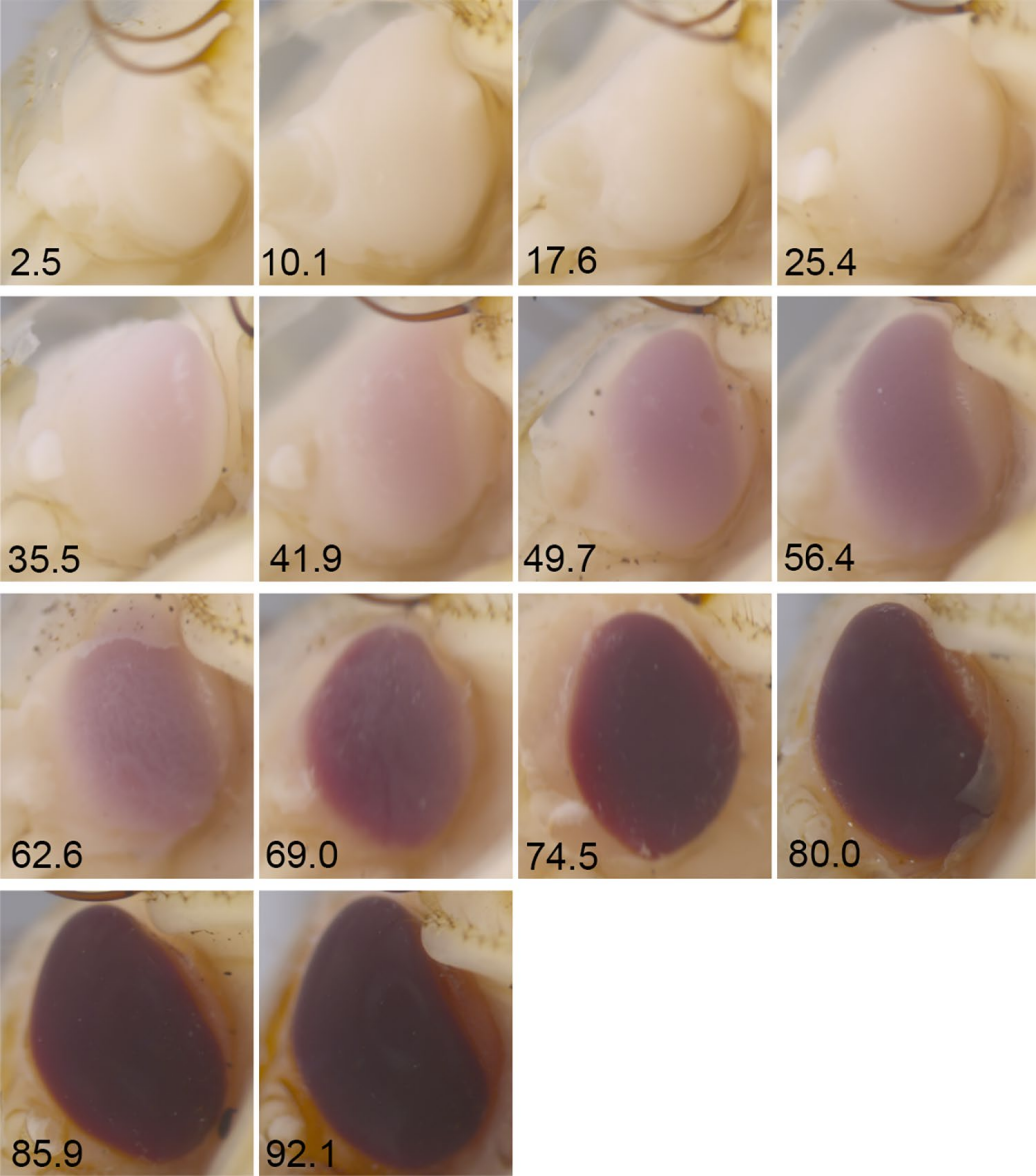
Changes in the pupal eye pigmentation along the *Necrodes littoralis* pupal development timeline in 20 °C. Numbers are for pupal age (ADD above 9 °C).

**Figure 6:**
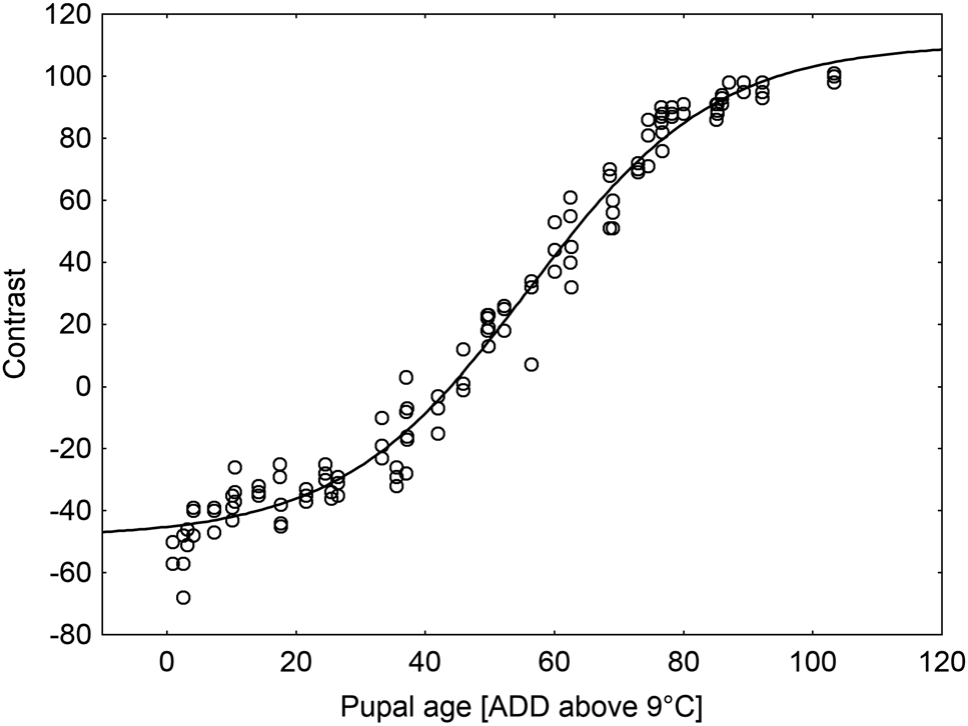
The model for the relationship between eye-background contrast and the pupal age (ADD above 9 °C) of *Necrodes littoralis*.

Eye pigmentation of pupae reared across different temperatures became gradually darker along the pupal development timeline (Fig. 5). As a consequence, eye-background contrast increased with the pupal age, initially at a low rate, then (after about 30 ADD) at a high rate and finally (after about 90 ADD) at a low rate again (Fig. 6). The model for the relationship was:

$$Contrast=-48.5871+ \frac{110.5042 -\left(-48.5871\right)}{1+46.2122{e}^{-0.0685Age}}$$

with highly statistically significant estimates of all parameters (*P* < 0.001; Table 1; for the dataset used to build the model see supplementary material) and the fit of 98% (Fig. 6).

**Table 1:** Estimates of model parameters.

Parameter	Estimate	*SE*	*t*	*P*	Confidence interval
*A*	− 48.5871	2.32353	− 20.9109	< 0.001	− 53.1880;− 43.9863
*K*	110.5042	4.09712	26.9712	< 0.001	102.3915;118.6169
*Q*	46.2122	11.07481	4.1727	< 0.001	24.2829;68.1414
*B*	0.0685	0.00459	14.9159	< 0.001	0.0594;0.0776

### Validation of the method

**Figure 7:**
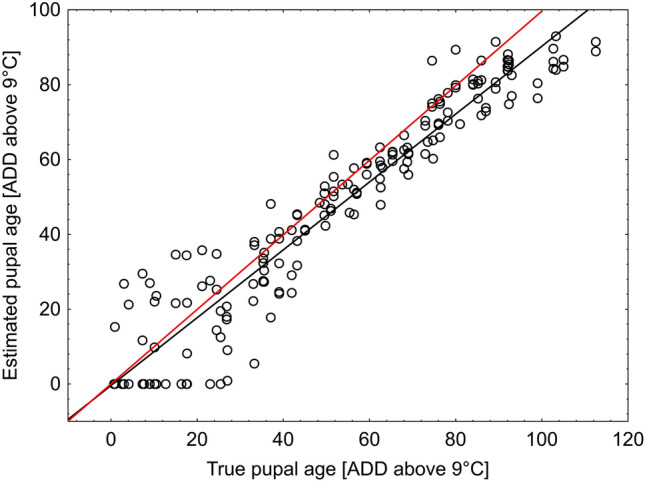
Estimated pupal age plotted against true pupal age (ADD above 9 °C). Black line—regression model for the relationship between true and estimated pupal age. Red line represents perfect estimates.

**Figure 8:**
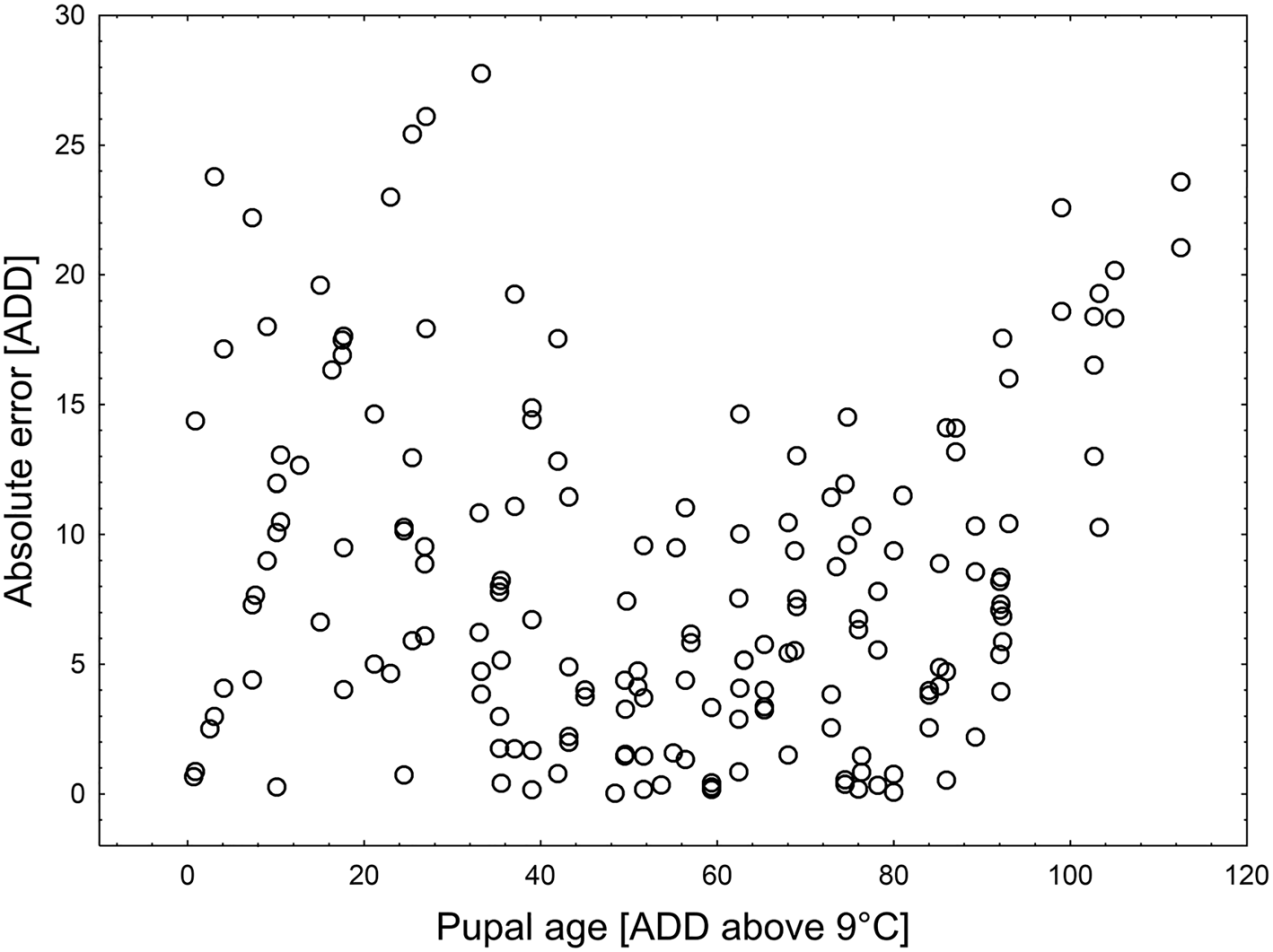
Absolute error of pupal age estimation (ADD) along with the true pupal age (ADD above 9 °C).

**Figure 9:**
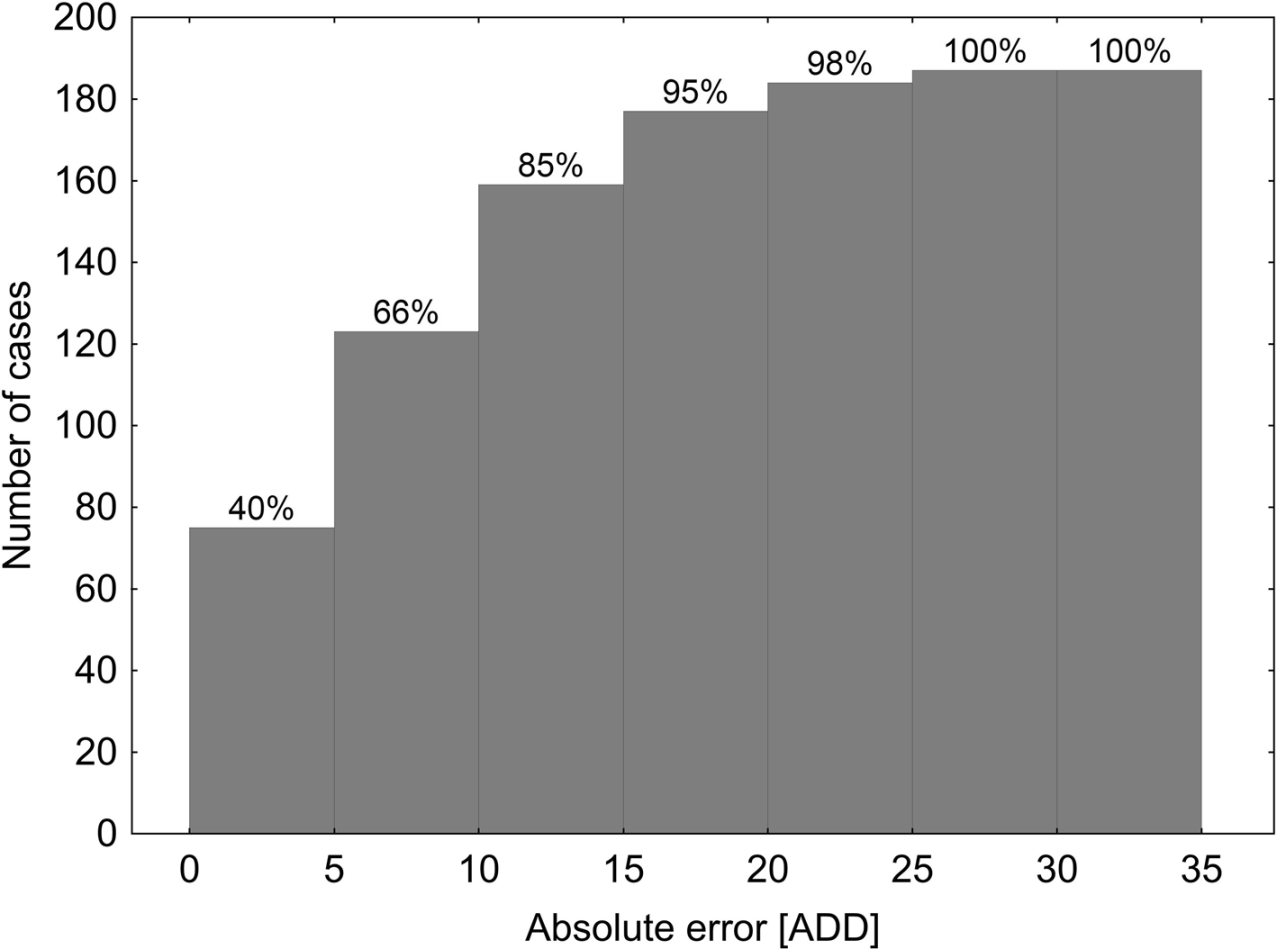
Cumulative distribution of absolute errors in pupal age estimation (ADD).

In general, the method slightly underestimated pupal age (Fig. 7). It was less accurate in case of very young pupae (until about 30 ADD) as well as mature pupae (after about 90 ADD, Figs. 7,8). The mean error was 8.12 ADD and the largest error was 27.8 ADD. Moreover, 95% of estimates deviated from the true age by no more than 20 ADD and 66% of estimates had error lower than 10 ADD (Fig. 9).

**Figure 10:**
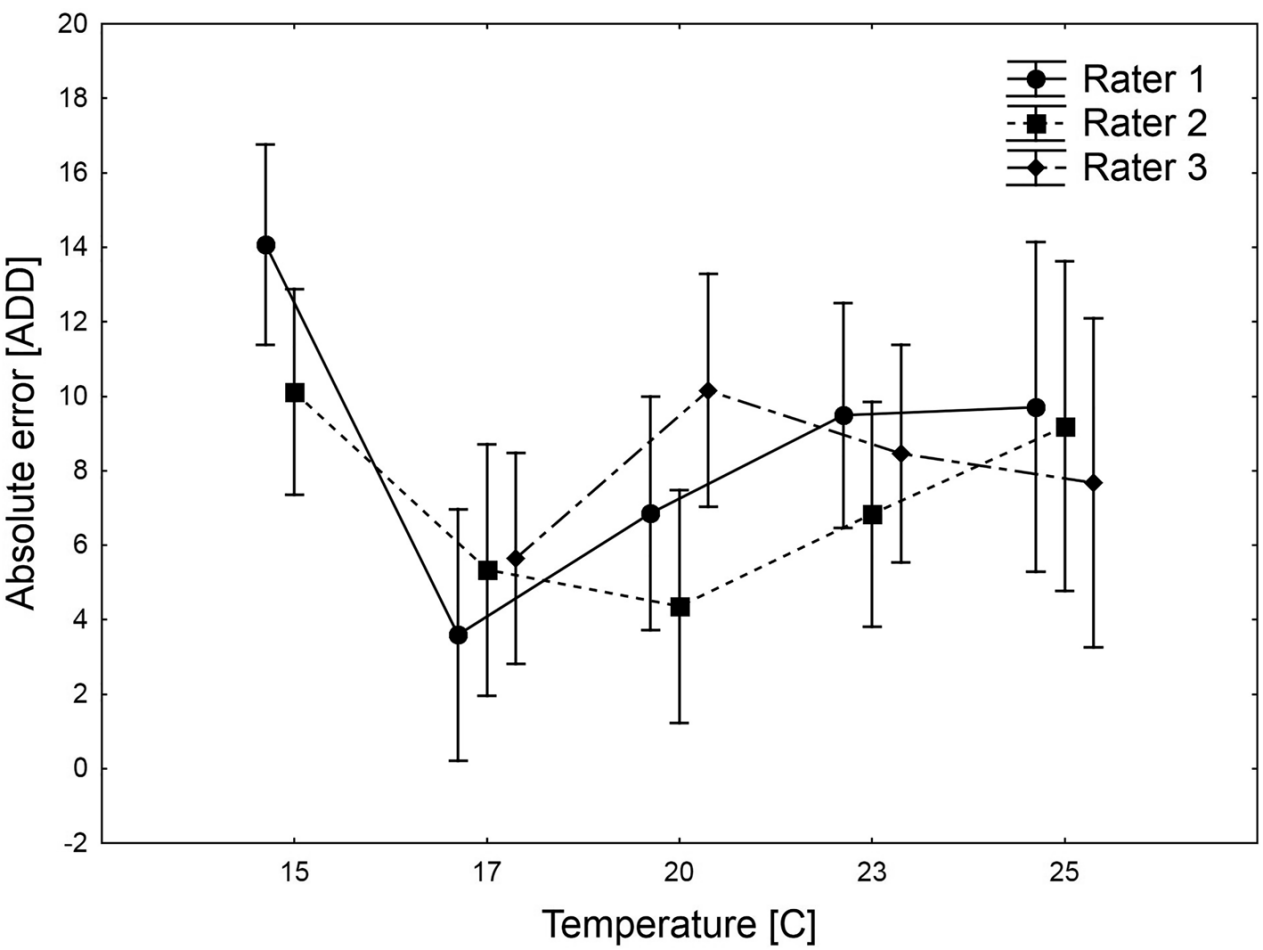
Differences in absolute error of pupal age estimation between the raters and the rearing temperatures. Symbols are means, vertical lines are 95% confidence intervals for the means.

While using the method, different raters attained similar accuracy, but we found significant differences in the error rate between the temperatures, the interaction was insignificant (two-way ANOVA, rater factor: *F*_2, 138_ = 0.85, *P* = 0.43, temperature factor: *F*_3, 138_ = 3.31, *P* = 0.022, interaction: *F*_6, 138_ = 1.18, *P* = 0.32; Fig. 10).

## Discussion

Eye-background contrast revealed clear advantages as a pupal age marker in case of *Necrodes littoralis*. First, its increase was gradual, and its changes covered most of the pupal stage, although in the initial and final portions of the stage it changed at a lower rate. Secondly, its use for pupal age estimation was associated with a relatively low error rate. The average error was about 8 ADD (above 9 °C) or about half of a typical summer day and 95% of age estimates deviated from the true age by no more than 20 ADD (above 9 °C) or about a day and a half during the typical summer. Thirdly, the contrast measurement technique, as proposed in this article, may be regarded as reliable. Different raters estimated pupal age with similar accuracy, attaining consistent values across the whole pupal development timeline and for pupae reared under different temperature and rearing conditions. The technique, therefore, proved to be resistant to human and setup-environment factors. Morever, by quantifying contrast between the eye and the background (instead of the measurement of eye pigmentation only), it was possible to eliminate artefactual effects of different equipment, different lighting conditions etc. when different entomologists will use the technique. We assume that the differences in intensity between the eye and the background (i.e. contrasts) are largely resistant to the noise generated by all the possile differences in the setup. However, before the external validation of the method, we recommend to keep the setup as close as possible to the one used in this study. Fourthly, the technique is simple, fast and cheap in comparison to other techniques for pupal age estimation. For instance, contrary to morphological techniques, it may be used by an entomologist with no expert skills in pupal morphology. Apart from a basic stereomicroscope with a camera, and a PC with simple graphic software, no other equipment is necessary to use the method.

The significant advantage of the technique is its likely universality. The majority of intra-puparial development studies of flies included the eye color of a pupa^22,36–38,44,45,59^. When we investigated published pupal eye pictures^28,29,38^ we found strong support for the prediction that our technique will work effectively with forensically useful flies, as well. Method validation is thus needed in general, particularly in case of blow flies and flesh flies.

There are several minor disadvantages of our method. During the initial and final portions of the pupal stage, changes in contrast were smaller. As a consequence, young and old pupae were aged with lower accuracy than middle-aged pupae. Moreover, the method is unsuitable for live specimens, as it is partly destructive. A single antenna and epidermis covering the eye need to be removed. The rest of the specimen may, however, still be used for other analyses. Insect preservation techniques were found to affect specimen appearance and therefore may influence pupal eye pigmentation^39,57^. These problems may be eliminated, if pupae sampled on a death scene and pupae that were used to derive published contrast data are preserved in the same way. In this study, we killed pupae by immersion in near-boiling water and preserved them in 75% alcohol, which is in accordance with standard forensic practices^3,24,57^. However, when death scene pupae are killed or preserved with different techniques, the use of reference data from this study may result in less accurate estimates of the pupal age. Since the eyes may whiten over time, the interval between preservation and analysis of the specimen should not be longer than necessary.

The technique may be improved. As with every development-based entomological method, increase in sampling frequency during laboratory studies improves the quality of resultant reference data and eventually accuracy of the age estimation method^60^. The current model was developed based on daily sampling of pupae. We recommend that future studies use 12 h or even smaller sampling intervals throughout the pupal stage. Because eye-background contrast changed at a lower rate during the initial and final portions of the pupal stage, another possibility for improvement would be to combine contrast with some other quantitative trait. The multi-trait technique would be most effective, if contrast was supplemented with a trait that changes in the initial and final portions of the pupal stage.

Another possible modification would consist of photographing the background together with the eye in a single picture. This would lower the risk of accidental change in light conditions between the pictures and would make the technique faster (exposure would have to be adjusted only once, in a single picture). However, a single picture has also clear disadvantages, which may outweigh the advantages. First, the background may be covered by parts of the pupa or its holder that are out of the focus. Second, the pupa usually casts some shadow on the background. These interferences would affect RGB values for the background and consequently would lower precision of the contrast quantification using our technique.

## Materials and methods

### Rearing and preparation of pupae

Pupae of *Necrodes littoralis* were reared in constant temperature conditions of 17, 20 and 23 °C using temperature chambers (ST 1/1 BASIC or + , POL EKO, Poland). Postfeeding third instar larvae from our main colony (20–23 °C, insect rearing containers, 30 males and 30 females, humid soil, pork meat ad libitum) were transferred to the soil-filled Petri dishes (3 larvae/dish) and their postfeeding and pupal development was monitored while being kept in the chamber. Larvae used in the experiments came from egg batches oviposited by different females. Multiple batches were used per temperature. The pupal development was asuumed to start at the moment when a postfeeding larva molted into a pupa. The moment may be recognized easily, as the pupa is white and immediately visible when the molting starts and upon inspection it was usually fully emerged. Petri dishes were inspected usually twice a day, on some occasions they were checked every 2–4 h to collect very young pupae. If a pupa was recorded, we assumed that molting started in the midpoint between the current and the previous inspection. The error was no more that 6 h, and sometimes much less. Pupae were sampled usually once a day and sometimes more frequently. In total, there were 12 sampling time-points at 17 °C, 14 at 20 °C and 23 °C. At each time-point three pupae were sampled, providing 36 pupae for 17 °C, 42 pupae for 20 °C and 23 °C. Pupae were killed and fixed by immersion in near-boiling water for 5 min, then left on a paper tissue for another 5 min to dry out and cool down and subsequently stored in 75% EtOH. The specimens were analyzed within a maximum of several days after preservation. Transparent pupal epidermis covering the eye and the adjacent antenna (or both antennae) was removed with fine tweezers and insect pins before pictures were taken. Pupae are stored in Laboratory of Criminalistics (AMU, Poznań, Poland).

### Photographing

Specimens were stabilized in a plastic holder placed on the middle grey photography card (the background), which was cropped to fit the immersion-container (Fig. 2b). The holder was fully submerged in 75% EtOH (Fig. 2c). A paper diffuser was used to prevent light artefacts (Fig. 2d). Pictures of eyes in lateral view were taken using Leica DFC 450 camera mounted on Leica M165C stereomicroscope and with three external lamps (Fig. 2d). Microscope and camera settings were controlled using Leica Application Suite, v 4.1.0.

For each specimen, pairs of pictures were taken with the same lighting conditions, magnification and camera settings. A picture of the eye was taken first, then the container was gently moved and when the grey background filled the whole screen, a picture of the background was taken with the same camera, microscope and light settings. To avoid extreme values of intensity, RGB intensity-histograms were checked while taking pictures of the background and if necessary, exposure was adjusted for both pictures.

An sRGB color palette (sRGB IEC61966-2.1) was used for the camera and the software.

### Exposure adjustment

To unify exposure within (and across) the picture pairs, each background image was opened in Adobe Photoshop CS2 and its intensity was displayed using the histogram tool (*Window* → *Histogram*) and the RGB channel (Fig. 3). Because the background pictures displayed only the grey photography card, analyses and adjustments were made on the entire picture. The mean RGB intensity of each background was changed to meet 150 value by individually adjusting the *Exposure* bar for each image (in *Image* → *Adjustments* → *Exposure*) until the predefined intensity value (i.e. 150) has been reached (Fig. 3b). The resultant exposure-change factor was subsequently applied to adjust exposure in the corresponding eye image (Fig. 3).

### Contrast quantification

RGB intensity was measured in the largest possible elliptical area of the pupal eye using the *Elliptical Marquee Tool* of the Adobe Photoshop CS2 (Fig. 4a). Mean RGB intensity in the area was quantified using the *Histogram* tool (Fig. 4b). Eye-background contrast for each pair of images was calculated by subtracting the mean RGB intensity of the eye from the mean RGB intensity of the background (in all cases 150, 149.5–150.5). The contrast values for each pair of images were used in subsequent analyses.

### Model for the relationship between contrast and pupal age

Pupal age was expressed in accumulated degree-days (ADD). To calculate ADD we used 9 °C as a base temperature (*T*_*b*_)^61^ and a formula $ADD=\left(T-{T}_{b}\right)\times age$, where *T* is the rearing constant temperature in °C and *age* is the pupal age in days. After inspecting scatter plot, we chose the generalized logistic function to model the relationship between pupal age (ADD) and contrast. The function is:

$$Contrast=A+ \frac{K -A}{({1+Q{e}^{-BAge})}^{1/v}}$$

where *A* and *K* are respectively lower and upper asymptote, *B* is the growth rate, *Q* is the value of the predictor variable (here *Age*) at which the response variable (here *Contrast*) is 0 and *v* shows whether the growth is larger near the lower or upper asymptote. After data inspection, we decided to fix *v* at 1 and as a result, we had four parameters to estimate from the data (*A*, *K*, *B* and *Q*). The function is:

$$Contrast=A+ \frac{K -A}{1+Q{e}^{-BAge}}$$

Parameters were estimated using Levenberg–Marquardt procedure in Statistica 13 (TIBCO Software Inc., US).

### Validation study

To test the method, we performed a validation study with pupae reared at constant temperatures of 15, 17, 20, 23 and 25 °C and contrast measured by three raters (entomologists: female 28 years old, female 33 years old, male 43 years old). Pupae from 15 °C were analyzed by two raters. Pupae were sampled at 19 time-points in 15 °C, 12 time-points in 17 °C, 14 time-points in 20 °C, 15 time-points in 23 °C and 7 time-points in 25 °C (five pupae per time-point). Pupae were analyzed one to three moths after their preservation, depending on the temperature and the rater. Each rater analyzed different pupae from a given time-point. Protocols for rearing and preparation of pupae, photographing, exposure adjustment and contrast quantification were the same as during the main part of the study. In total, 182 specimens were analyzed. Pupae are stored in the Laboratory of Criminalistics (AMU, Poznań, Poland).

Contrast measurements were used to estimate pupal age using the transformed function:

$$Age=\frac{1}{B}\mathrm{ln}\left(\frac{Q(Contrast-A)}{K-Contrast}\right)$$

The estimated age was compared against the true age to determine the error rate of the method. To test the significance of differences in error rate between temperatures and raters we performed a two-way ANOVA with rater factor considered at 3 levels and temperature factor considered at 4 levels (without 15 °C for which only two raters analyzed pupae). All calculations were made in Statistica 13 (TIBCO Software Inc., US).

## Supplementary information

Supplementary information

## Data Availability

The dataset analyzed to build the contrast model was provided as a supplementary online material. The other datasets generated and/or analyzed during the study are available from the corresponding author on a reasonable request.
